# The Population Density, Interspecific Territoriality, and Philopatry of Shrikes (Laniidae) and Bushshrikes (Malaconotidae) in a Mosaic of Natural and Human-Modified Acacia Savanna

**DOI:** 10.3390/biology14050577

**Published:** 2025-05-20

**Authors:** Grzegorz Kopij

**Affiliations:** Department of Vertebrate Ecology, Wrocław University of Environmental & Life Sciences, ul. Kozuchowska 5b, 51-631 Wroclaw, Poland; grzegorz.kopij@upwr.edu.pl

**Keywords:** population density, competition, behavioural ecology, *Eurocephalus auguitimens*, *Laniarius atrococcineus*, *Nilaus afer*, *Dryoscopus cubla*, *Tchagra australis*

## Abstract

In avian behavioural and spatial ecology, issues related to population density, territoriality, and philopatry are especially important. These are often interrelated. The aim of this study was to analyse these behaviours in shrikes inhabiting an acacia savanna in northern Namibia (400 ha). Population densities of one shrike and four bushshrike species were studied by means of the territory mapping method. About half of the study area had been modified by humans into cultivated fields, orchards, sport fields, and human settlements. This study was conducted in 2012/2013, 2017/2018, and 2019/2020. The most common species were the Black-backed Puffback (2.5 pairs/100 ha) and Crimson-breasted Shrike (1.9); the least common were the Southern White-crowned Shrike (0.8) and Brubru (1.0). Statistics for the Brown-crowned Tchagra indicated a density of 1.5 pairs/100 ha. While the density of the Brown-crowned Tchagra and Brubru remained stable over the years, the population densities of the Black-backed Shrike, Crimson-breasted Shrike, and Southern White-crowned Shrike showed a remarkable increase over the years 2012–2020. All bushshrike species showed a preference for patches of natural savanna vegetation. This was especially evident in the Crimson-breasted Shrike and the Brubru. However, the Southern White-crowned Shrike from the shrike family did not show this preference. The presented studies showed marked interspecific differences in territoriality and philopatry. Furthermore, even within the same species, marked temporal differences were shown in population density, probably also in philopatry. Within families, these behaviours may shape both resource competition and mate competition, while between families, these behaviours only shape resource competition.

## Simple Summary

Population density, habitat preference, territoriality, and philopatry are important aspects of avian ecology. These aspects were studied in shrikes inhabiting a mosaic of natural and human-modified savanna in Namibia. Marked differences in population density and philopatry were shown among species. However, the levels of population density, interspecific territoriality, and philopatry differed temporally, even within the same species. These variations are probably adaptive responses to resource competition and reproductive interference.

## 1. Introduction

Ecology can be considered the experimental analysis of the distribution and abundance of species [1]. Distribution and population density have been successfully determined in various vertebrates (e.g., [2]). With regard to birds, such analyses have been thoroughly conducted in Europe, resulting in a large amount of data [3,4,5,6,7]. These are, however, still far from completion in the tropical regions of the world [8]. For example, in southern Africa, whose fauna is one of the most intensively studied among the tropical regions of the world, bird distribution has been well researched [9,10,11]; still, little is known about the abundance of many species there, especially those from the order Passeriformes. Even population densities of conspicuous and strongly territorial birds, such as shrikes (Laniidae) and bushshrikes (Malaconotidae), belonging to this order, are poorly investigated [9,12]. These groups are well represented in Africa, with 136 species from the family Malaconotidae (excluding Prionopidae and Platysternidae, as today these are regarded as separate families) and 31 species from the family Laniidae [12]. In Namibia, ten and seven species, respectively, have been recorded [13].

Shrikes are also known to be strongly territorial species. Since they comprise a diverse group in Africa and many species are common, they are a convenient object of studies on territoriality. The phenomenon of territoriality has been intensively studied in many Palearctic and Nearctic bird species [3,4,6], but much less frequently studied in the Afrotropical region [9,12]. Territoriality usually has an intraspecific character, and its main function is population density regulation [1]. There are, however, cases of interspecific territoriality, even among species that are not closely related, for example, between *Parus major* Linnaeus 1758 and *Fringilla coelebs* Linnaeus 1758 in Scotland [14]; *Acrocephalus* species in Europe [5,6,7,8,9,10,11,12,13,14,15,16,17]; *Corvus corone* Linnaeus 1758–*C. monedula* Linnaeus 1758–*C. frugilegus* Linnaeus 1758 in the UK [18]; *Lanius collurio* Linnaeus 1758–*L. nubicus* Lichtenstein 1823 in Egypt [19]; *Asio otus* (Linnaeus 1758)–*Megascops asio* (Linnaeus 1758)–*Aegoluis accadicus* (Gmelin 1788) in Michigan, USA [20]; *Strix varia*–*Tyto alba* (Scopoli 1769) in Michigan [20]; and Parulidae in North America [21]; among *Sylvia*; and among *Phylloscopus* species in the UK and Scandinavia [22]. Drury et al. [23] have shown that interspecific territoriality is widespread, having been recorded in 32% of Nearctic passerines, most of which (73%) are territorial with only one other species, but with 19% of cases involving species that are not closely related, i.e., represented by different families. Knowledge on the issue of interspecific territoriality and philopatry in African birds is almost non-existent [9,12]. This paper partly fills this gap in our knowledge.

The following hypotheses have been tested in the presented study: (1) shrikes and bushshrikes breed in higher population densities in natural savanna than in human-modified savanna; (2) they achieve higher densities in years with higher precipitation than in years with lower precipitation; (3) due to similar diet preferences, particular shrikes and bushshrikes show territoriality in relation to other shrike and bushshrike species; and (4) bushshrikes show high levels of philopatry.

## 2. Materials and Methods

### 2.1. Study Area

The study area is situated on the Ogongo UNAM Campus, Omusati Region, N Namibia (17°40′ S, 15°17′ E). The Cuvelai Drainage System, where the study area is located, is a unique ecosystem comprising a network of natural water canals (oshanas), mopane and acacia savannas [24,25,26]. The natural vegetation comprises the acacia savanna composed mainly of *Vachellia erioloba*, *Acacia nilotica*, *Senegallia fleckii*, *S. mellifera*, *Albizia anthelmintica*, *Dichrostachys cinerea*, *Colophospermum mopane*, *Combretum* spp., *Commiphora* spp., *Grewia* spp., *Ficus sycomorus*, *Boscia albitrunca*, *Sclerocarya birrea*, *Terminalia sericea*, *Zyzyphus mucronata*, and *Hyphaene petersiana* [27]. There is only a small part of the mopane savanna, composed almost entirely of young *Colophospermum mopane* shrubs. Both savannas are utilised as a pasture for the cattle, sheep, and goats.

The total surface of the study area was 400 ha. Most of it (70%) constitutes a natural acacia savanna, and the remaining has been converted into yards with buildings, arable fields, orchards and sport fields (Table 1). There are also numerous exotic trees planted in and around human settlements, such as *Kigelia africana*, *Moringa oleifera*, *Melia azedarach*, *Dodonaea viscosa*, and *Eucalyptus camelduensis.* There are several permanent water bodies, and the area is bordered with an artificial water canal to the north and with an extensive oshana (natural grassy depression filled with water in the rainy season) to the east.

**Figure 1:**
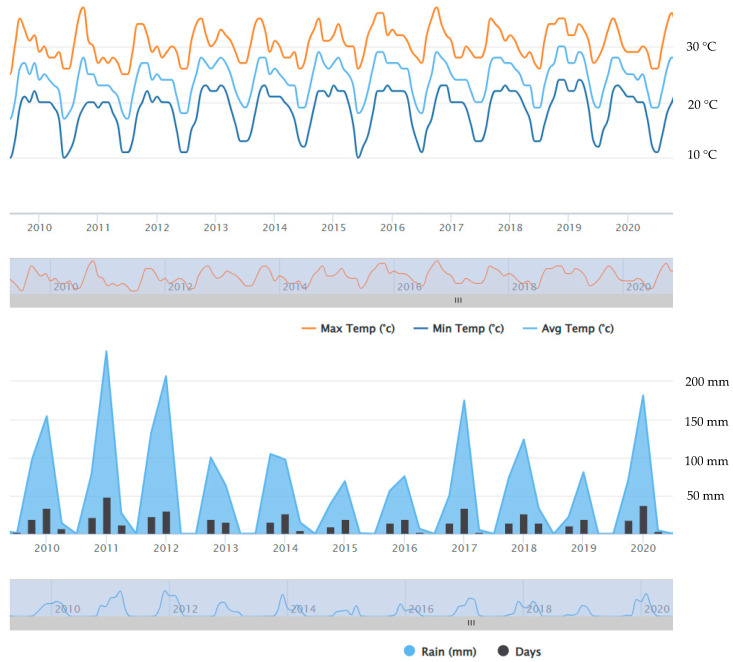
Average monthly temperature (**above**) and quarterly rainfall (**below**) in Onguadiva in 2009–2020. https://www.worldweatheronline.com/ongwediva-weather-averages/oshana/na.aspx, accessed on 5 May 2025.

**Figure 2:**
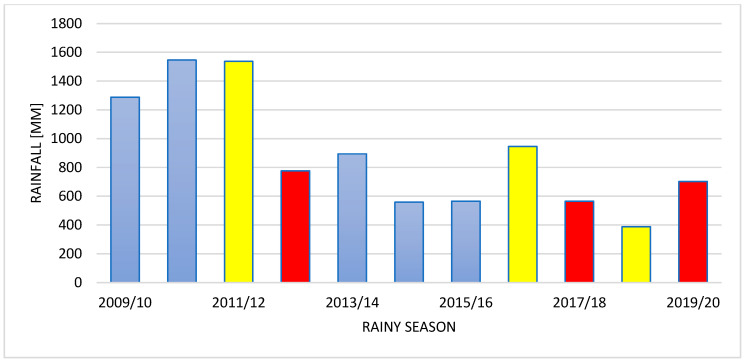
Year-to-year changes in the amount of rain in rainy seasons (September–April) in Onguadiva from 2009–2020. Red columns indicate rainy seasons during which studies were conducted; yellow columns—rainy seasons preceding these studies; blue columns—the remaining rainy seasons.

Ogongo has a semi-arid climate. Summers are sweltering and partly cloudy while the winters are short, comfortable and clear [24,26]. The mean temperature varied slightly over the years 2009–2020 (Figure 1). The total amount of rain in Onguadiva was 702 mm in 2019/2020 rainy season (September–April) and 388 mm in the preceding rainy season (Figure 2); the rainfall was only 565 mm in 2017/2018 rainy season, whereas it was much higher (946 mm) in the preceding rainy season. The amount of rain was 776 mm in the 2012/2013 rainy season, but it was very high (1537 mm) in the preceding 2011/2012 rainy season. In brief, the amount of rainfall was much more varied from year to year than the average temperature in this area (Figure 1 and Figure 2) (https://weatherandclimate.com/namibia/oshana/ongwediva, accessed on 5 May 2025).

### 2.2. Methods

Studies were conducted in three rainy seasons: 2012/2013, 2017/2018, and 2019/2020. The 2012/2013 rainy season was characterised by an exceptionally high precipitation followed by two years of similar high precipitation. On the other hand, both 2017/2018 and 2019/2020 rainy seasons were characterised by a low precipitation. The 2017/2018 rainy season was preceded by a rainy season with a higher precipitation, while the 2019/2020 rainy season had lower precipitation (Figure 1). These rainy seasons were selected to show the effect of the rainfall on the population density, interspecific territoriality, and philopatry.

The territory mapping method [28,29] has been applied to assess population densities of shrike (Laniidae) and bushshrike (Malaconotidae) species breeding in the study area. The territory mapping method is considered one of the most accurate methods for estimating bird population densities and for studying population dynamics and subtle relationships between birds and their breeding habitat. In this method, a breeding pair, not an individual, is the census unit. In this method, it is assumed that an occupied territory is equal to one breeding pair, although polygamy or co-operative breeding occurs sometimes and may slightly distort results.

Most bird species are territorial during the breeding season. Usually males are engaged in establishing and defending territory boundaries. Males also advertise their territories with songs. If a bird species is uncommon, the area is not completely filled with its territories, leaving some ‘open’ spaces. In this case, mapped registrations of birds of a given species, showing territorial or breeding behaviour, will fall into clusters. The cluster indicates occupied territory, and this in turn is assumed as being an equivalent of one breeding pair. If a bird species is abundant, it has closely packed territories without ‘free’ space. In this case, mapping simultaneously singing males is especially important to distinguish territories. To increase detectability of occupied territories and to increase the accuracy of the population density estimation, counting should be repeated over the whole study area several times in the breeding season. Each count/survey should be separated by at least two-week intervals. In this method, an occupied territory (=breeding pair) is therefore used as the census unit.

In this study, four surveys of the whole area were conducted in each rainy season (October–May) by one and the same researcher. Field observations were aided with ZEISS 10 × 50 binoculars (Oberkochen, Germany) and GARMIN eTrex 20 × tourist GPS (Olathe, KS, USA). In the 2012/2013 rainy season, the first survey was conducted in the first half of November, the second survey in the second half of November, the third survey in the first half of December, and the fourth one in the second half of December. In the 2017/2018 rainy season, the first survey was conducted in the first half of February, the second survey in the second half of February, the third survey in the first half of March, and the fourth one in the second half of March. In the 2019/2020 rainy season, four surveys were conducted in each of the three following seasons: February/March, April, and May/June.

Since the study plot was too large to survey in one morning, 4–5 morning (each morning lasted from 5–6 to 9–10 a.m.) counts were required to complete the survey of the whole study plot. Birds calling (especially important were singing birds) and showing other territorial or breeding behaviour were identified to species and plotted on the map. Noted were the number of birds observed and the kind of performed behaviour. Caution was taken to not register the same individuals by noting movements of birds in the field and by paying special attention to birds calling at the same time. At least two records of a given species in a clump were required to distinguish an occupied territory [29]. However, if a nest with eggs or chicks were found, one record was sufficient. Most of these nests have not been accessed, and most were discovered while fledglings were present around the nests. 

### 2.3. Statistical Analysis

The population density was expressed as the number of breeding pairs per 100 ha. The dominance in the community of shrikes and bushshrikes was defined as the percentage of breeding pairs of a given shrike or bushshrike species in relation to the total number of all breeding pairs of all shrike and bushshrike species.

The study plot consisted of a mosaic of natural and transformed acacia savanna habitat. In order to measure selectivity of natural versus transformed habitats, each mapped territory was classified into three groups: (a) covering mostly (>75%) or entirely natural habitat; (b) covering mostly (>75%) or entirely transformed habitat; (c) covering approximately equal proportions of natural and transformed habitats.

The degree of the philopatry was calculated as the proportion between the number of territories held in the same or partly the same site to the number of territories held in different sites in two compared rainy seasons, i.e., 2012/2013 vs. 2017/2018, 2012/2013 vs. 2019/2020, and 2017/2018 vs. 2019/2020. The proportion was expressed as a percentage.

Year-to-year differences in population densities were tested with the *χ*^2^ test. The relationship between the precipitation and number of breeding pairs of a particular species was examined with correlation analysis.

## 3. Results

**Figure 3:**
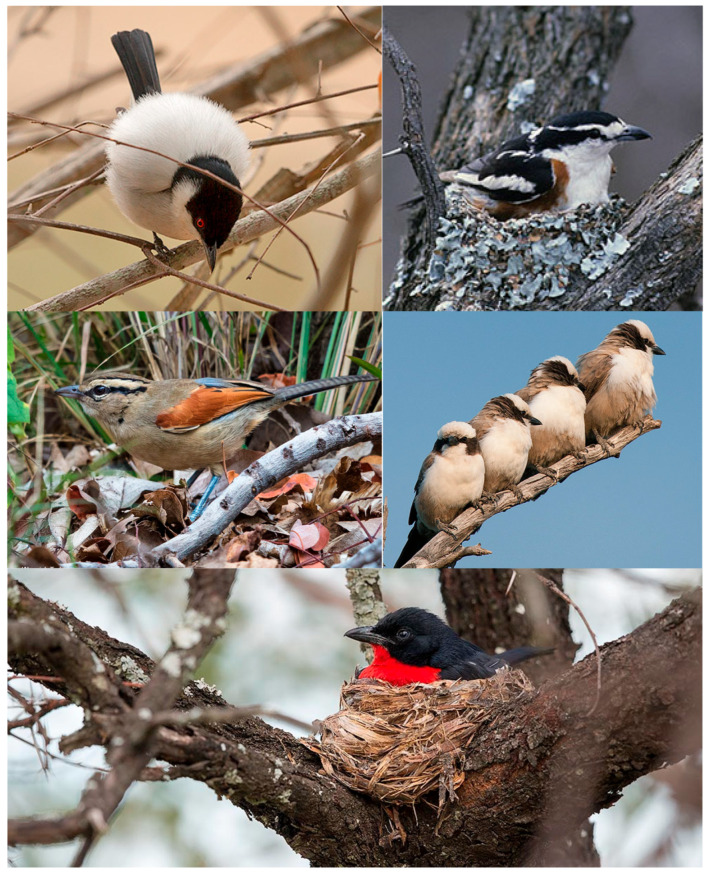
Upper left: Black-backed Puffback; upper right: Brubru; middle left: Brown-crowned Tchagra; middle right: Southern White-crowned Shrike; lower: Crimson-breasted Shrike. All photographs by W. Tarboton.

One shrike and four bushshrike species were recorded as breeding in the study plot: Southern White-crowned Shrike (*Eurocephalus auguitimens* Smith 1836) from the family Laniidae and four species from the family Malaconotidae: Crimson-breasted Shrike (*Laniarius atrococcineus* (Burchell 1822)), Brubru (*Nilaus afer* (Latham 1801)), Black-backed Puffback (*Dryoscopus cubla* (Latham 1801)), and Brown-crowned Tchagra (*Tchagra australis* (Smith 1836)) (Figure 3). The population densities of each species ranged from 0.3 to 2.5 pairs per 100 ha; it was the highest in the Crimson-breasted Shrike. The Crimson-breasted Shrike dominated the shrike/bushshrike community in each rainy season; the Black-backed Shrike dominated in 2017/2018 and in 2019/2020; while the Brown-crowned Tchagra was dominant in 2012/2013 only (Table 2).

While the population density of the Brown-crowned Tchagra and the Brubru remained stable, that of the Black-backed Shrike, Crimson-breasted Shrike, and Southern White-crowned Shrike showed a remarkable increase over the years 2012–2020 (Table 2).

All bushshrike species showed a preference for patches of natural savanna vegetation. This was especially evident in the case of the Crimson-breasted Shrike and the Brubru. However, the Southern White-crowned Shrike, from the shrike family (Laniidae), did not show this preference (Table 3). Most Crimson-breasted Shrike, Black-backed Shrike, and Black-crowned Tchagra territories were situated in the same or nearly the same sites in the 2012/2013, 2017/2018, and 2019/2020 rainy seasons (Table 4), which suggest a fairly high degree of philopatry. Due to a low sample size these differences are, however, not significant statistically.

**Figure 4:**
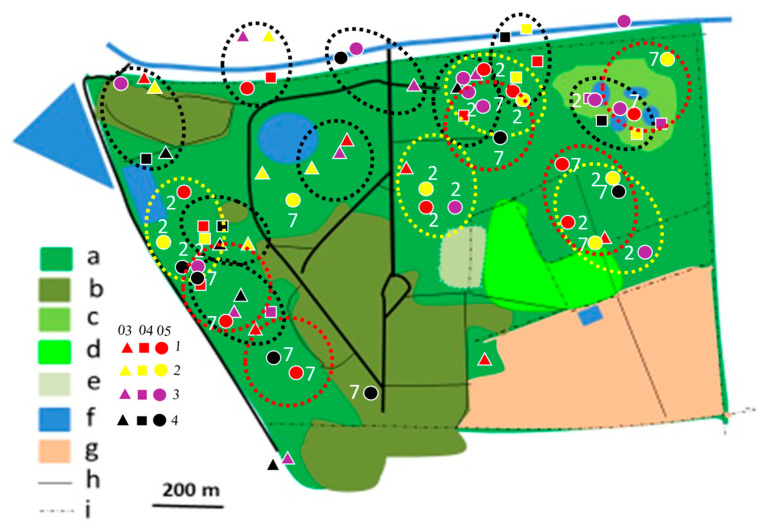
Distribution of Crimson-breasted Shrike territories in Ogongo in 2012/2013, 2017/2018, and 2019/2020 rainy seasons. Explanations: small triangles, quadrates, and dots indicate mapped registrations during surveys 1, 2, 3, and 4. In 2012/2013 and 2017/2018 four surveys were conducted (all indicated with dots). In 2019/2020 four surveys were conducted in February/March (03, triangles), four in April (04, quadrates), and four in May/June (05, dots) (12 surveys altogether). At least two records in one clump were required to delineate occupied territory. Encircled are occupied territories in 2012/2013 (yellow dotted circles with registrations denoted as ‘2’), 2017/2018 (red dotted circles with registrations denoted with ‘3’), and 2019/2020 (black dotted circles with registrations not denoted with figures). Explanations of habitats (land uses): a—acacia savanna, b—built-up area, c—disturbed acacia savanna, d—orchard, e—sport field, f—water bodies, g—arable ground, h—roads, i—fences.

**Figure 5:**
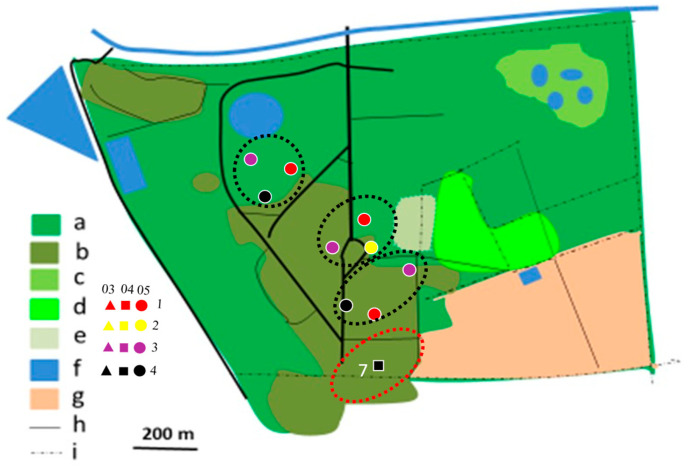
Distribution of Southern White-crowned Shrike territories in Ogongo in 2017/2018 (yellow dotted circles) and 2019/2020 (black dotted circles) rainy seasons. No territory was established in 2012/2013 rainy season. Explanations as in Figure 4.

**Figure 6:**
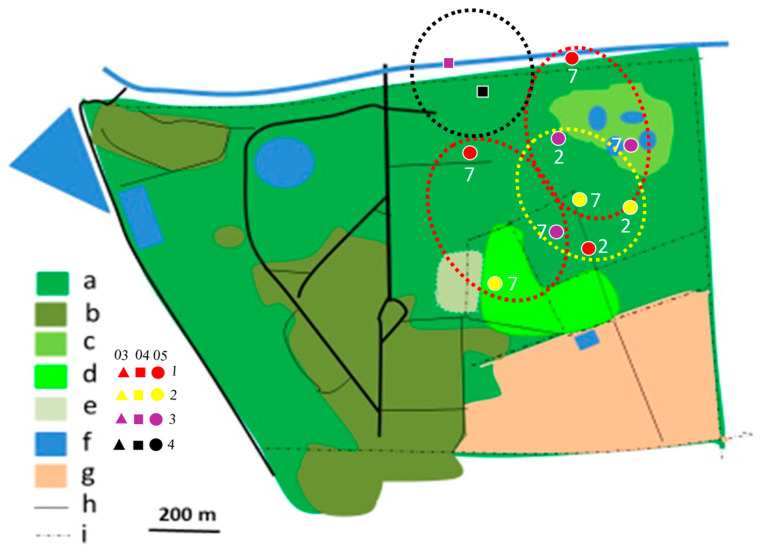
Distribution of Brubru territories in Ogongo in 2012/2013 (yellow dotted circles), 2017/2018 (red dotted circles), and 2019/2020 (black dotted circles) rainy seasons. Explanations as in Figure 4.

**Figure 7:**
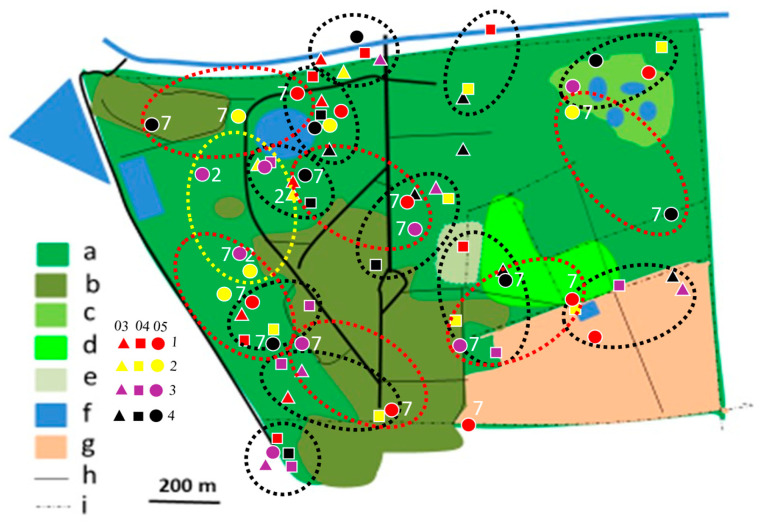
Distribution of Black-backed Puffback territories in Ogongo in 2012/2013 (yellow dotted circles), 2017/2018 (red dotted circles), and 2019/2020 (black dotted circles) rainy seasons. Explanations as in Figure 4.

**Figure 8:**
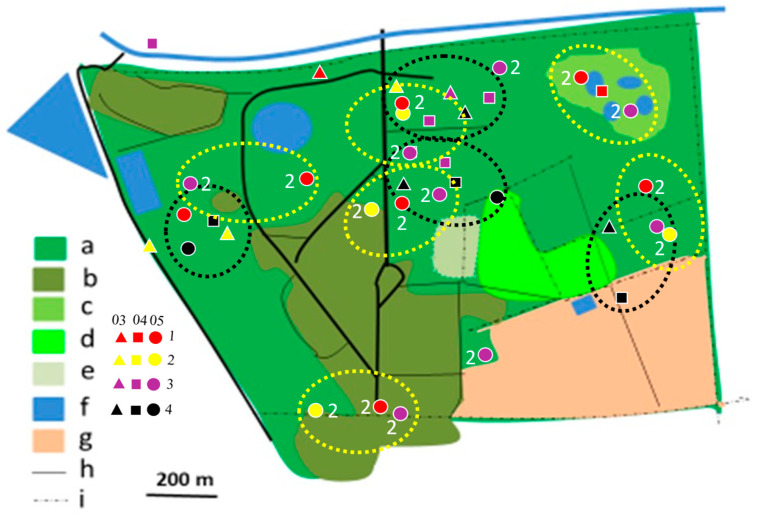
Distribution of Brown-crowned Tchagra territories in Ogongo in 2012/2013 (yellow dotted circles) and 2019/2020 (black dotted circles) rainy seasons. No territories were established in 2017/2018 rainy season. Explanations as in Figure 4.

**Figure 9:**
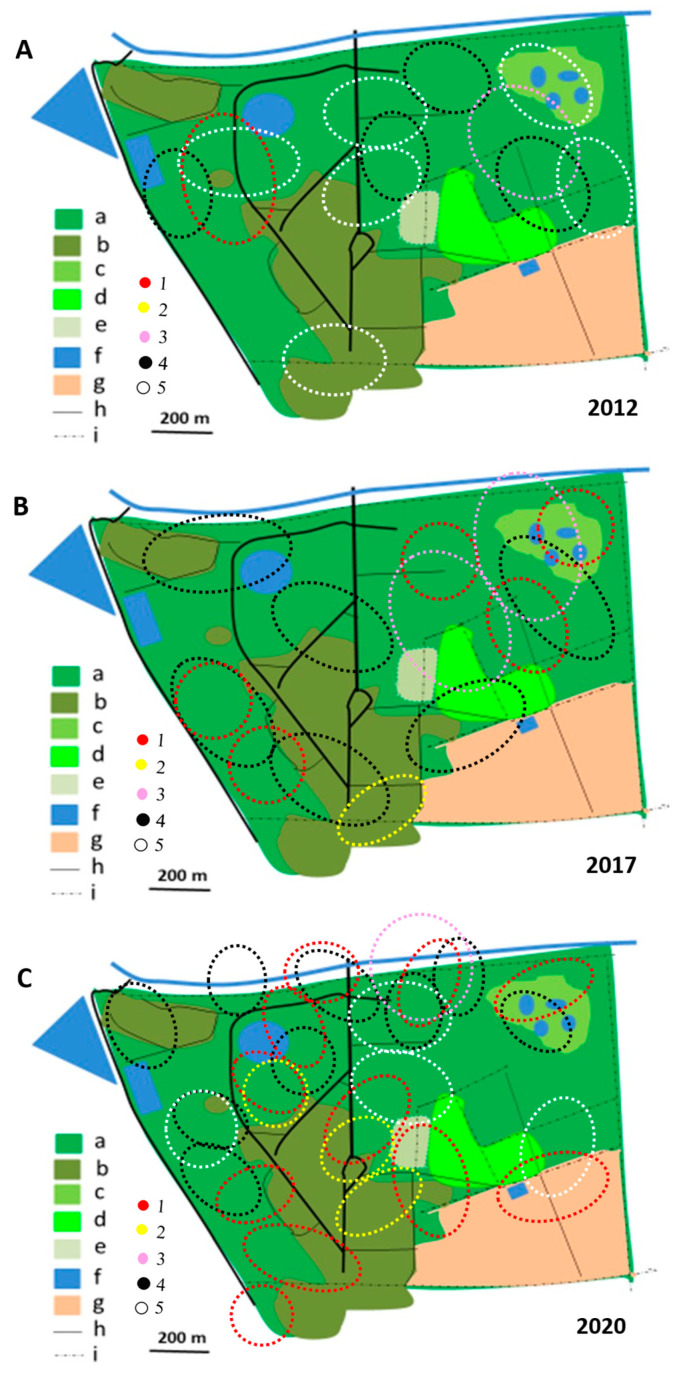
Shrike and bushshrike territories in (**A**)_2012/2013, (**B**) 2017/2018 and (**C**) 2019/2020 rainy seasons, as delineated in Figure 4, Figure 5, Figure 6, Figure 7 and Figure 8. Explanations of species: each dotted circle indicates occupied territory: 1 (red)—Black-backed Puffback, 2 (yellow)—Southern White-crowned Shrike, 3 (pink)—Brubru, 4 (black)—Crimson-breasted Shrike, 5 (white)—Brown-crowned Tchagra. Explanations of habitats (land uses): a—acacia savanna, b—built-up area, c—disturbed acacia savanna, d—orchard, e—sport field, f—water bodies, g—arable ground, h—roads, i—fences.

In 2012/2013, most territories of the Brown-crowned Tchagra and the Crimson-breasted Shrike remained separated, while only single territories of two other species were established (Figure 4, Figure 5, Figure 6, Figure 7, Figure 8 and Figure 9). In the 2017/2018 rainy season, most territories of the Black-backed Puffback excluded Crimson-breasted Shrike territories. The Brubru territories partly overlapped with the Black-backed Puffback territories but excluded Crimson-breasted Shrike ones (Figure 9). The Southern White-crowned territory excluded territories of all other shrikes and bushshrikes (Figure 5). In the 2019/2020 rainy season, the overall population density of shrikes and bushshrikes in the study area was twice that in 2012/2013 and 2017/2018 rainy seasons (Table 1). Crimson-breasted Shrike territories excluded six Black-backed Puffback territories, but the remaining five territories overlapped to a lesser or greater extent (Figure 7 and Figure 9). Brown-crowned Tchagra territories completely excluded White-crowned Shrike territories (Figure 8 and Figure 9). Territories of the latter species overlapped with those of the Black-backed Puffback but excluded territories of the Crimson-breasted Shrike (Figure 9).

**Figure 10:**
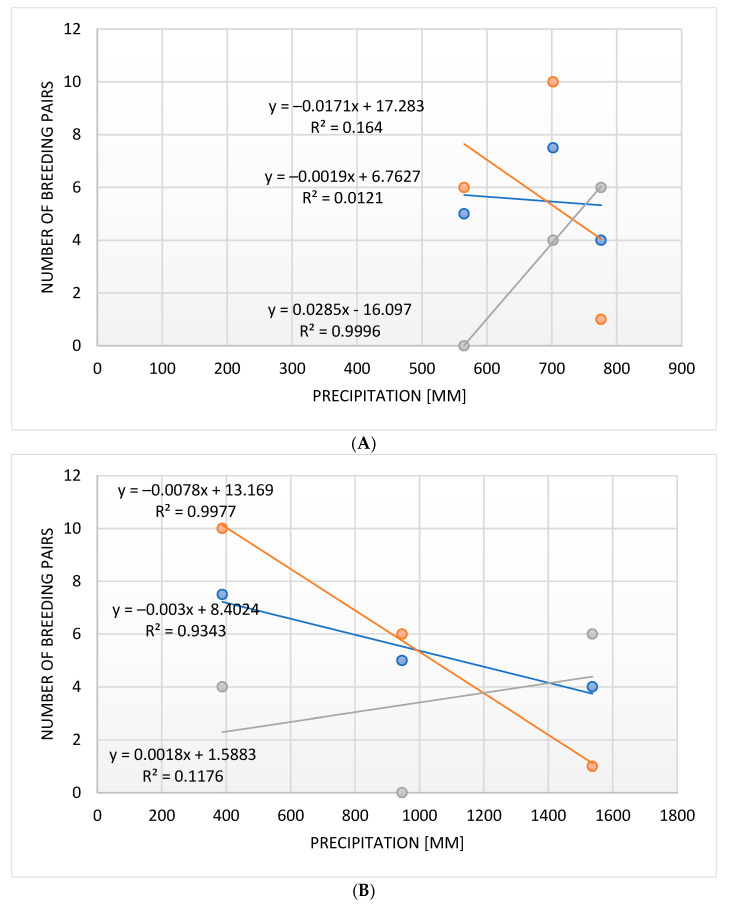
The correlation between the number of breeding pairs and the precipitation in the breeding seasons (**A**) and in the years preceding these (**B**) in three bushshrike species (orange: Black-backed Puffback, blue: Crimson-breasted Shrike, grey: Brown-crowned Tchagra).

A strong negative correlation has been recorded between the amount of rain in a given breeding season and the number of breeding pairs of the Brown-crowned Tchagra, but no such correlation was recorded for the Crimson-breasted Shrike and the Black-backed Puffback (Figure 10). This negative correlation in these two species was, however, very strong regarding the amount of rainfall in the preceding breeding season and the number of breeding pairs, while no correlation was recorded in the Brown-crowned Tchagra (Figure 10).

## 4. Discussion

Data on population densities of African shrikes and bushshrikes are available only from one biome, woodland [9]. As expected, the population densities are, overall, higher in woodland than in savanna (this study). This remains true in regard to particular species occupying both biomes. A probable reason for these differences in population densities is the fact that woodland is more productive than savanna in the same geographic region [30]. In the ecotone zone (woodland/savanna), the densities could be even higher than in woodland. However, shrike and bushshrike population densities have not been studied so far in ecotone zones.

In broad-leaved woodland in the province of Limpopo, South Africa, the Brubru nested at a density of 2–3 pairs/100 ha [31], which is higher than the density recorded in this study (Table 1) or in Kasane, NE Botswana [32]. The Black-backed Shrike nested at a density of 2.4 p./100 ha. It is therefore similar to that recorded in this study (2.5 p./100 ha) and in Kasane (3.0 p./100 ha [32]). The Brown-crowned Tchagra reached a density of 4 p./100 ha, which is much higher than in this study (1.0 p./100 ha) and in Kasane (1.3 p./100 ha [32]). On the other hand, the Crimson-breasted Shrike nested at a density of 8 p./100 ha, which is much higher than in present study (1.9 p./100 ha).

Therefore, in Ogongo only the Black-backed Puffback had a population density comparable to that recorded in natural woodland in Limpopo [31]. Other bushshrikes in woodland reached much higher densities compared with savanna in Ogongo. As shown in this study, bushshrike species show a strong preference for savanna, most of which has been modified or transformed, hence their population densities were lower compared with woodland in Limpopo [31].

Population densities of bushshrikes are much higher in acacia savanna than in neighbouring mopane savanna (Table 4). The densities of the selected shrike and bushshrike species are also much higher in savanna than in grasslands (Table 5). In grasslands two other shrike species, the Common Fiscal (*Lanius collaris* (Linnaeus 1766)) and Bokmakierie (*Telophorus zeylonus* (Linnaeus 1766)), are common and sometimes even dominant in avian communities [33,34,35]. The only bushshrike species which appears to not be affected by habitat alternations is the Black-backed Puffback. The highest population density recorded for this species was in the urbanised habitat of Kasane [31].

The population densities of shrikes and bushshrikes may be influenced by other species present in the avian community. In the study area in Ogongo, there were about 110 other breeding bird species, including seven coraciiform, four raptor, and four owl species [36,37]; G. Kopij, own data). Their feeding niches may partly overlap with those of the shrikes and bushshrikes, and some raptors may also prey on shrikes and bushshrikes. Perhaps the Black-backed Puffback’s preference for nesting in human-altered habitats is an adaptation to avoid some of these predacious birds, which may, on the other hand, avoid human-altered habitats. The other explanation is that the invertebrates upon which the shrike preys are more common in human-modified habitats.

Territoriality is often accompanied by philopatry, which can be defined as an attachment to the natal place. It can be assumed that the stronger the territoriality, the higher the degree of philopatry. Philopatry may affect the territorial behaviour, mortality rate and reproductive success, population density and dynamics, and genetic variability of a population [38]. The degree of philopatry is not only variable interspecifically but may also vary within a species. It is shaped by environmental factors and population density [39]. Philopatry is, however, understudied and the subject is especially little known in African birds [9,12].

Interspecific territoriality in birds is expected: (a) between species that prey on large, ground-dwelling arthropods, from a vantage point; (b) between ground-foraging species; (c) between raptors preying on small mammals; (d) between species foraging on tree bark [40]. Shrikes and bushshrikes belong to the first group, as their diet includes large ground-dwelling invertebrates and small vertebrates hunted from a perching site. Unfortunately, no quantitative data are available on the diet composition [9,12,41]. The presented study suggests the occurrence of interspecific competition between some of them. It is probably an adaptive response to resource competition and reproductive interference. Within families, it may shape both resource competition or mate competition, while between families it may shape resource competition only [23].

The presented study suggests that shrikes and bushshrikes show relatively high philopatry (Table 3). In the study area, many territories were shifted to other places, from year to year, but this may not necessarily indicate low philopatry. The degree of philopatry may be even higher than the overlapping territories indicate (Figure 6). The applied method is, however, not best suited to studying philopatry, as particular individuals are not recognisable. Also, sexes are not distinguishable in these birds due to the lack of sexual dimorphism. More robust data can be obtained with colour-ringed birds.

Little is known about philopatry in shrikes and bushshrikes [41]. In the Lesser Grey Shrike (*Lanius minor*), a long-distance migrant, 30% (97/319) of the nests were built in the same nest tree in successive years and more than half (183/319 = 57.4%) of the nests were in the same or neighbouring trees (up to 20 m). However very seldom were the nests built by the same individuals [38]. In the Loggerhead Shrike (*Lanius ludovicianus*) in North America, 14% of adult birds were philopatric; this figure was probably low because of high winter mortality [42]. In two sympatric migratory shrike species in Japan, the Bull-headed Shrike (*Lanius bucephalus*) and Brown Shrike (*L. cristatus*), philopatry was low in the former (18% of males, 0% of females) but high in the latter species (43% of males and 13% of females) [43]. In this study, the highest philopatry was recorded in the Black-backed Puffback (75.0%), Brown-crowned Tchagra (66.7%), and Crimson-breasted Shrike (61.5%), but it was much lower (33.3%) in the Brubru, and there was none in the Southern White-crowned Shrike (Table 4). It should be pointed out, however, that the sample size in the present study was much lower than in the abovementioned studies.

Takagi [43] suggested that philopatry should be higher in habitat specialists compared to habitat generalists. Weatherhead and Forbes [44] evidenced, on the other hand, that migratory passerines are characterised by a low natal philopatry compared to resident passerines. Zimmerman and Finck [45] suggested that the degree of philopatry is related to the previous reproductive success of a particular pair: the higher the reproductive success the higher the philopatry. The shrikes in the presented study can be classified as food specialists. They prey mostly on larger arthropods collected from the ground. Being restricted mainly to acacia savanna, they are all residents [9,12,41]. It is therefore not surprising to find that shrikes and bushshrikes displayed in this study a relatively high level of philopatry.

**Table 5:** Population densities (pairs/100 ha) of shrike and bushshrike species in Ogongo compared to other sites in Namibia.

Species	A	B	C	D	E
Southern White-crowned Shrike	0.8	0.2	0	0	0
Crimson-breasted Shrike	1.9	0.5	0	0	0.3
Brubru	0.0	0.4	1.2	0	<0.1
Black-backed Puffback	2.5	0.2	1.2	5.0	0
Brown-crowned Tchagra	1.0	0.7	0	0	<0.1

A: Ogongo campus, acacia savanna, north-central Namibia, 400 ha, this study; B: Ogongo Game Park, mopane savanna, 3000 ha, [36]; C: Katima Mulilo, Zambezi riparian forest, Zambezi Region, NE Namibia, 2014/15, 85 ha, [46]; D: Katima Mulilo, urbanised habitat (data pooled from 4 study plots; 476 ha), 2013–2015, [35,46,47]; E: Windhoek, central Namibia, urbanised habitat, 2014–2019, 50 km^2^, [48].

## 5. Conclusions

Shrikes and bushshrikes as strongly territorial birds constitute convenient objects of study on population density, territoriality, and philopatry. The presented study shows marked interspecific differences in these behaviours. Overall, the most common in the study were the Crimson-breasted Shrike and the Black-backed Shrike. Shrikes and bushshrikes show a strong preference for natural vegetation. A strong negative correlation has been recorded between the amount of the rainfall (in the current or the preceding rainy season) and the population density of the Brown-crowned Tchagra, Crimson-breasted Shrike, and the Black-backed Puffback. Results suggest that the strongest competition exists between these three species. Furthermore, even within the same species, marked temporal differences were shown in population density and philopatry. These variations are probably adaptive responses to resource competition and reproductive interference. High philopatry recorded in bushshrikes can be used in studies related to population dynamics and habitat selection, as well as resistance to habitat alteration. Further studies, with marked individuals, are required to better elucidate these aspects of shrike behavioural ecology in different habitats.

## Figures and Tables

**Table 1:** Microhabitats distinguished in the study area.

Microhabitat	Size [ha]	%
Natural savanna	278	69.5
Transformed savanna	122	30.5
Arable fields	30	7.5
Orchards	10	2.5
Sport fields	2	0.5
Yards with buildings	70	17.5
Total	400	100

**Table 2:** Population densities (pairs/100 ha) of shrike and bushshrike species in Ogongo. n—number of breeding pairs, d—density (pairs per 100 ha).

Species	2012/2013		2017/2018		2019/2020		*χ*^2^ Test		
	n	d	n	d	n	d	*χ* ^2^	df	*p*
Southern White-crowned Shrike	0	0	0.5	0.13	3	0.75	3.54	1	<0.05
Crimson-breasted Shrike	4	1	5	1.25	7.5	1.88	1.18	2	>0.5
Brubru	1	0.25	2	0.50	0.5	0.13	1.00	2	>0.5
Black-backed Puffback	1	0.25	6	1.50	10	2.50	7.18	2	<0.05
Brown-crowned Tchagra	6	1.5	0	0	4	1.00	5.40	1	<0.05
Total	12	3.0	13.5	3.38	25	6.26	6.01	2	<0.05

**Table 3:** The number of territories of shrike and bushshrike species established in the natural vs. transformed savanna.

Species	Natural Savanna		Partly Natural/Partly Transformed		Transformed Savanna	
	n	%	n	%	n	%
Southern White-crowned Shrike	1	0.33	1	0.33	1	0.33
Crimson-breasted Shrike	16	94.1	1	5.9	0	0.0
Brubru	3	100	0	0.0	0	0.0
Black-backed Puffback	5	62.5	2	25	1	12.5
Brown-crowned Tchagra	7	70	2	20	1	10

**Table 4:** The philopatry in shrikes and bushshrikes. The number of territories held in the same site (T), partly the same site (P), and in different sites (N) in two compared rainy seasons.

Species	2012 vs. 2017			2012 vs. 2020			2017 vs. 2020			Total		
	T	P	N	T	P	N	T	P	N	T	P	N
Southern White-crowed Shrike	-	-	-	-	-	-	0	0	1	0	0	1
Crimson-breasted Shrike	0	2	2	1	1	2	2	2	1	3	5	5
Brubru	1	0	0	0	0	1	0	0	1	1	0	2
Black-backed Puffback	0	1	0	0	1	0	3	1	2	3	3	2
Brown-crowned Tchagra	-	-	-	0	4	0	-	-	-	0	4	2
Total	1	3	2	1	5	3	5	3	4	7	12	12

## Data Availability

The study did not report any data.
